# Topographic numerosity maps cover subitizing and estimation ranges

**DOI:** 10.1038/s41467-021-23785-7

**Published:** 2021-06-07

**Authors:** Yuxuan Cai, Shir Hofstetter, Jelle van Dijk, Wietske Zuiderbaan, Wietske van der Zwaag, Ben M. Harvey, Serge O. Dumoulin

**Affiliations:** 1 Spinoza Centre for Neuroimaging, Amsterdam, Netherlands; 2 Experimental and Applied Psychology, VU University Amsterdam, Amsterdam, Netherlands; 3 Experimental Psychology, Helmholtz Institute, Utrecht University, Utrecht, Netherlands

**Keywords:** Perception, Extrastriate cortex

## Abstract

Numerosity, the set size of a group of items, helps guide behaviour and decisions. Non-symbolic numerosities are represented by the approximate number system. However, distinct behavioural performance suggests that small numerosities, i.e. subitizing range, are implemented differently in the brain than larger numerosities. Prior work has shown that neural populations selectively responding (i.e. hemodynamic responses) to small numerosities are organized into a network of topographical maps. Here, we investigate how neural populations respond to large numerosities, well into the ANS. Using 7 T fMRI and biologically-inspired analyses, we found a network of neural populations tuned to both small and large numerosities organized within the same topographic maps. These results demonstrate a continuum of numerosity preferences that progressively cover both the subitizing range and beyond within the same numerosity map, suggesting a single neural mechanism. We hypothesize that differences in map properties, such as cortical magnification and tuning width, underlie known differences in behaviour.

## Abstract (web summary)

Here, the authors show that the brain represents small and large numerosity ranges in a continuous topographic map, in line with the idea that differences in map properties underlie differences in perception.

## Introduction

Perception of numerosity (the set size of a group of items) guides human and animal behaviour and decisions^1–4^. Both humans and animals perceive numerosity over a wide numerical range. The approximate number system (ANS) is a core system that is commonly recognized to process non-symbolic number (i.e., numerosity) and relates to symbolic number processing^5,6^. The ANS is thought to produce an intuitive “number sense” across species^7^ and throughout human development^8^, and represent increasing numerosities with decreasing precision in accord with Weber’s law^9^. Primarily based on the distinct behavioural performances, a separate system termed object tracking system (OTS)^10^ is thought to process small numerosities, typically up to four, known as subitizing range^11^. This system is thought to be distinct from larger numerosities, known as estimation range^12^. Evidence supporting the distinct systems for numerosity processing comes from the discontinuous behavioural performances, such as reaction time and accuracy, and distinct neural signatures^13,14^. For example, numerosity judgements within the subitizing range yields accurate enumerations, which fails for larger numerosities, and may violate Weber’s law^15^.

However, the separate numerosity systems are not universally accepted^16–18^. Neurophysiological studies on non-human primates found neurons that selectively respond to different numerosities^19,20^. These numerosity-selective neurons respond to small and large numerosities with similar logarithmic tuning functions as human^21^. Single neuron recording studies conducted on monkeys and crows found no sudden change in the behavioural performance and no distinct neural responses between small and large numerosities^22,23^. Moreover, numerosity discrimination follows Weber’s law in both small and large numerosities^24,25^. Thus, these studies suggest that there is no need to assume separate systems for small and large numerosities.

Here we investigate the neural mechanisms underlying the representation of small and large numerosities in the human brain. We refer to the numerosity ranges as small and large, because subitizing range varies between participants and we did not tailor our experiment for individual participants^26^. We measured BOLD responses of numerosity-selective neural populations within functional magnetic resonance imaging (fMRI) recording sites^27^. We have previously shown these populations to respond maximally to numerosities in a small range (i.e., 1–7) and to be arranged in orderly topographic maps^28^. Here we measure their responses to a wider range of numerosities, well into the ANS (i.e., 1–64).

Based on prior knowledge about topographic maps^29,30^ and numerosity processing^31,32^, we will evaluate two hypotheses. First, small and large numerosities may be processed in distinct cortical regions. We have previously described neural populations responding maximally to small numerosities in an extensive network of topographically organized brain areas^27,28^. As perception of larger numerosities shows some different properties, such as more time-consuming and error-prone, larger numerosities may produce responses in distinct neural populations in a distinct set of areas. Second, neurons responding maximally to large numerosities could be placed in the same topographic map, i.e., along the systematic topographic progression including both the small and large ranges. This would be akin to stimulating greater eccentricities in the same visual field map^30^. Even if small and large numerosities are represented at the same topographic map, there may still be perceptual differences between small and large numerosities. For example, central versus peripheral vision are processed in the same topographic visual field map, but their perception differs considerably^30^. Following this hypothesis, neural populations responding to large numerosities may display distinct properties, such as broader tuning, thus leading to different perceptual properties.

We investigate these hypotheses using ultra-high field (7 Tesla) fMRI and population receptive field (pRF) modelling^33^. We measured BOLD response of neural populations that tuned to small and large numerosities and compared estimated neural numerosity preferences to investigate how different numerosity ranges are represented in the brain. We find that both numerosity ranges are represented in the same topographic maps, and we suggest that differences in neural response selectivity and topographic map properties, such as tuning width and cortical magnification respectively, underlie the different perceptual and behavioural properties of small and larger numerosities.

## Results

### Neural populations in the same cortical regions respond to small and large numerosities

**Fig. 1:**
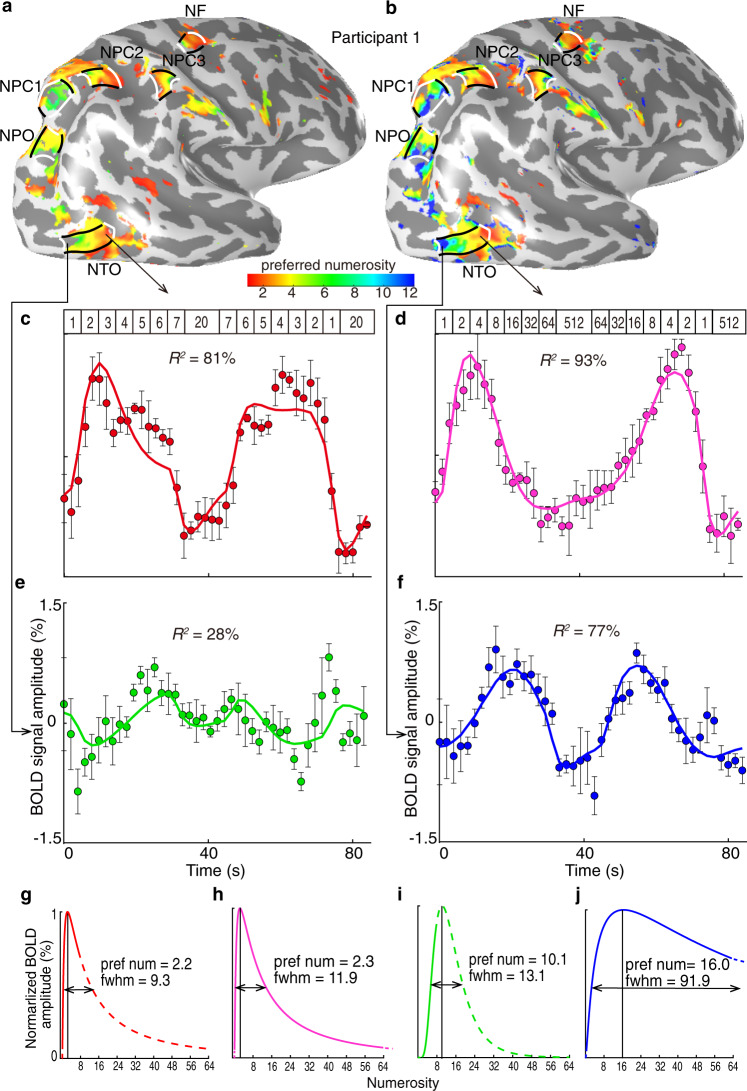
Neural population responses to small and large numerosities. **a**, **b** Cortical surface rendering of the right hemisphere shows a similar network of numerosity maps in both presented ranges. Preferred numerosities of cortical recording sites, estimated from responses to the small range (**a**) and the large range (**b**) for recording sites with over 30% of variance explained by the neural response model. Black lines outline the lateral borders of individual numerosity maps. The borders denoting the lowest and the highest preferred numerosities in each map are marked by white lines. An example fMRI recording site in anterior NTO shows different fMRI time courses (dots) for small (**c**) and large (**d**) numerosity ranges. Both time courses are similarly well captured by the predictions (coloured lines) of similar neural response models. Dots represent mean response amplitudes; error bars represent the standard errors over repeated measurements (*n* = 4). The presented numerosities are indicated at the top of the graph. **e**, **f** An example fMRI recording site in posterior NTO shows a higher preferred numerosity. This response does not reach a maximum in the small numerosity range (**e**). Dots represent mean response amplitudes; error bars represent the standard errors over repeated measurements (*n* = 4). **g**, **h** For both numerosity ranges, the anterior NTO site’s response is predicted by similar neural response models. **i**, **j** For the large numerosity range, posterior NTO site’s response is well predicted by a neural response model (**j**). However, this sites’ preferred numerosity is above the small numerosity range, so it could not be determined accurately (**i**), produces only low-amplitude responses and yields poorer model fits (**e**) with this range. Preferred numerosity is indicated by the highest response amplitude in the neural model, and tuning width is indicated by the full width at half maximum (FWHM). The neural response model within the presented range is shown with solid lines, outside the range with dashed lines.

When participants viewed the small numerosity range, i.e., 1–7, we found neural populations tuned to these small numerosities. These neural populations were organized in a network of topographic numerosity maps in line with our previous observations^27,28^. This network consisted of six numerosity maps, in the temporo-occipital cortex (NTO), parieto-occipital cortex (NPO), parietal cortex (NPC1-3), and in the superior frontal cortex (NF) (Fig. 1a, b). Within each map, the numerosity-selective neural populations changed gradually along the cortical surface in their preferred numerosity (the numerosity producing the largest response in each population). For example, in NTO (Fig. 1a), neural populations preferring smaller numerosities clustered at the inferior temporal gyrus while numerosity preferences increased posteriorly along the map (white lines). When participants viewed the large numerosity range, i.e., 1–64, we found a similar network of topographic numerosity maps as the one derived from viewing the small range (Fig. 1b). Similar networks of topographic numerosity maps were also found in all other participants Supplementary Fig. 1b.

To illustrate the tuned responses, we extracted the response time courses of two example recording sites (voxels) elicited by viewing the small (Fig. 1a, c, e) and large (Fig. 1b, d, f) numerosity ranges. These example sites are located in the anterior and posterior regions of the NTO map (Fig. 1a, b). For the anterior recording site, the neural response models captured more than 80% of the response variance in both conditions (Fig. 1c, d). This site had similar preferred numerosities in both conditions, i.e., 2.2 and 2.3, respectively (Fig. 1g, h). When viewing the small numerosity range, the posterior recording site’s response increased monotonically over the presented range, reflecting a preferred numerosity above 7 (Fig. 1i). However, this preferred numerosity could not be determined accurately as this response reached a maximum beyond the presented range (Fig. 1e). When viewing the large numerosity range, the maximum response occurred at the presentation of larger numerosities (above 7) (Fig. 1f). As this maximum was within the large stimulus range, this allowed us to determine the preferred numerosity at 16 (Fig. 1j). This demonstrates that neural populations with larger preferred numerosities are found near those with the small preferred numerosities at the same cortical area.

### Selectivity of neural populations remains stable

**Fig. 2:**
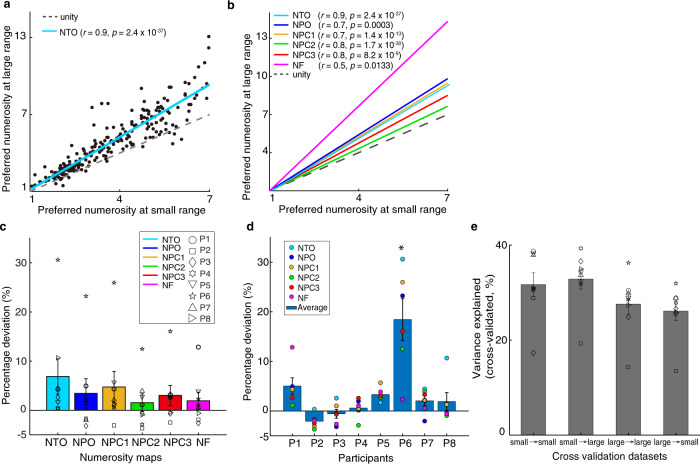
Relationship between numerosity preferences estimated from small and large ranges indicates similar numerosity selectivity and topographic progressions. **a** Participant 1’s NTO (see the maps in Fig. 1a & b) numerosity preferences estimated from the two ranges were strongly correlated (see legend of the Pearson correlation coefficients and statistical significance). Dots show the estimates from individual recording sites (variance explained >30%), the blue line shows the linear fit between the two estimates, the dashed line shows unity (i.e., identical preferences). **b** Linear fits from all six of this participant’s maps. These also reflect strong correlations in each map (see legends), indicating a similar spatial organization of estimated numerosity preferences, and are consistently above the unity line. **c** Bars show averaged percentage deviation quantifying the difference between the slopes of the linear fits (in (**b**)) and the unity line for each map. Error bars show the standard errors of the mean over participants (*n* = 8). A two-sided Wilcoxon signed rank test shows the percentage deviation of all these maps were above zero (*z* = 3.4, *p* = 0.0006, df = 47), suggesting a slight increase of preferred numerosity estimates at the large range. **d** Bars show averaged percentage deviation (same as in (**c**)) for each participant. Error bars show the standard errors of the mean (*n* = 6). Post hoc analysis shows significant difference between participant 6 and other participants (Bonferroni corrected for multiple comparions; * indicates *p* = 3.0 × 10^−8^). **e** Bars represent averaged cross-validated variance explained of the within- and cross-condition cross validation datasets. Error bars indicate standard errors of the mean over participants (*n* = 8). Within-subject two way ANOVA analysis shows no significant differences between the cross validation datasets (*p* > 0.025, two-sided, Bonferroni corrected for multiple comparisons). Source data are provided as a source data file.

We found strong correlations between the preferred numerosities estimated from the two numerosity ranges, especially for the overlapping portion (Fig. 2a, b), in all maps and all participants (Supplementary Fig. 2). We selected these preferred numerosities estimates based on two criteria: variance explained exceeded 30% and the preferred tuning fell within the presented ranges (i.e., 1–7 and 1–64 for the small and large ranges, respectively). This indicates a similar spatial organization of numerosity preferences between the two conditions, though it does not test how similar these preferences are. To quantify their similarity, we computed the extent to which the distribution of preferred numerosities estimated from the small and large ranges deviated from the unity line (where the two estimates are identical), i.e., the percentage deviation, for each map in each participant (see Methods). Zero percentage deviation indicates identical preferred numerosity estimates between conditions. A Wilcoxon’s signed rank test showed that the percentage deviations of all the maps across participants were significantly above zero (two-sided, *p* = 0.0006, *z* = 3.4, df = 47) (Fig. 2c). This demonstrated that preferred numerosities were significantly larger when estimated from the large numerosity range. However, the median percentage deviation was only around 3.59%, far smaller than the change in mean presented numerosities (454%), so, though significant, the effect size is small. ANOVA analyses of the percentage deviations in all the maps and participants demonstrated a significant effect of participant, but no effect of map and no interaction. Post-hoc analysis showed that only one participant had a significantly different percentage deviation from other participants (two-way ANOVA; F_(7,47)_ = 13.36, *p* = 3.0 × 10^−8^, followed by post hoc analysis, Bonferroni corrected for multiple comparisons) (Fig. 2d).

Furthermore, we performed a cross validation analysis (see “Methods”). To estimate the model’s predictability and reliability, we fit pRF estimates on one half split dataset to the response elicited by the other half split dataset and computed the cross-validated variance explained (i.e., cvR^2^) of the two conditions, respectively (within-condition cross validation). Next, we fit pRF estimates on small numerosity to the response elicited by large numerosity and computed the cvR^2^, and vice versa (cross-condition cross validation). We use the format of “pRF predictor → test data” (e.g., “large → small”) to indicate using data from large numerosity range to predict data acquired while viewing small numerosity ranges. We averaged the cvR^2^ from all the possible iterations: “small → small”, “small → large”, “large → large” and “large → small” cross validations, respectively. We then performed a within-subject two-way ANOVA analysis to compare the cvR^2^ between within- and cross-condition validations. There were no significant differences (*p* > 0.025, two-sided, Bonferroni corrected for multiple comparisons). As Fig. 2e shows, all of the half-split datasets show considerably high predictive power, suggesting that the pRF estimates are similar across conditions. The results of cross validation analyses also show strong correlations between preferred numerosity estimated from the two ranges and a slight increase of numerosity preference at the large range. (Supplementary Fig. 5a, b).

### More cortical area devoted to smaller numerosities

**Fig. 3:**
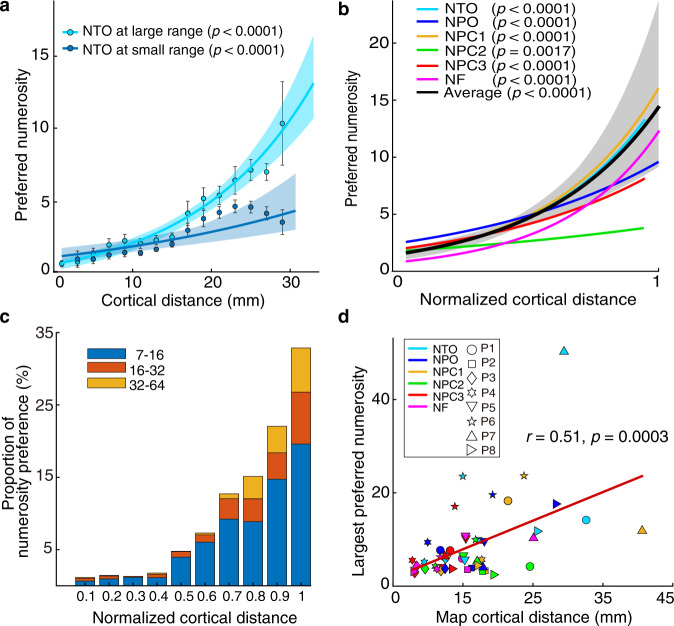
Visualization of the large numerosity preference locations. **a** Cortical progression of small (dark blue) and large (light blue) preferred numerosities with the cortical distance (between the white lines in Fig. 1a & b) across participant 1’s NTO map. The preferred numerosity increased systematically for both conditions. Points represent the mean preferred numerosity in each distance bin (every 2 mm); error bars showing standard errors of the mean over data points within each bin. Coloured lines show the best logarithmic fits. **b** Progression of numerosity preferences estimated from the large range as a function of normalized cortical distance in all the numerosity maps of participant 1. The black line shows the best logarithmic fit that bins the data points from all the maps across normalized cortical distance. Shade area shows the 95% confidence interval determined by bootstrapping fits (*n* = 1000) to the binned points and *p* values indicate the probability of the observed change from permutation analysis (*n* = 10,000), in both (**a**) and (**b**). **c** Proportion of tuned responses to large preferred numerosities (above 7) for each 10% interval of normalized cortical distance in all maps of all participants. Coloured bars represent the proportion of preferred numerosities ranging from 7 to 16, 16 to 32, and 32 to 64. **d** Map size (cortical distance) correlates with the largest preferred numerosities in the maps, i.e., large maps typically contain larger numerosity preferences. Source data are provided as a source data file.

The change of numerosity preferences along each map was quantified by measuring the distance of each data point from the borders of the map with the highest and the lowest numerosity preferences (white lines in Fig. 1a, b, see “Methods”). The numerosity preference progressed systematically along the cortical surface (Fig. 3a). Consistent with previous studies^27,28^, we found a cortical magnification effect, with less cortical surface responding to larger numerosities, in all the maps of all the participants (Fig. 3b, Supplementary Fig. 3).

To visualize the location of populations with large numerosity preferences (above 7), we calculated the proportion of large numerosity preferences in each 10% cortical distance interval. As shown in Fig. 3c, neural populations tuned to large numerosities are located towards the end of the maps. This suggests that numerosity preferences progressed from small to large continuously along the same topographic map. Last, we found a significant correlation between the size of the maps (cortical distance) and the largest preferred numerosity in these maps (*r* = 0.51, *p* = 0.0003; Fig. 3d). This suggests that tuned responses to larger numerosities are more detectable in larger maps. Using cross validation datasets, similar systematic progressions were found across all maps and all participants (Supplementary Fig. 5c).

### Tuning width increases with preferred numerosity

**Fig. 4:**
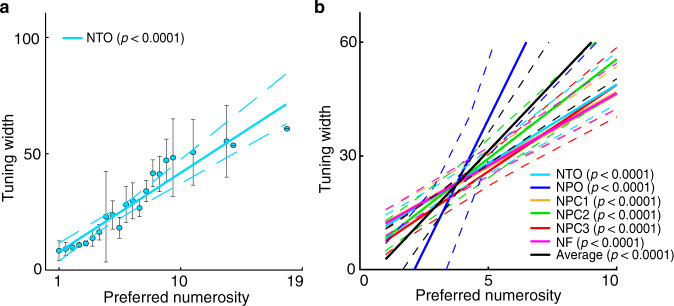
Tuning width changes with preferred numerosity. **a** Tuning width increases with preferred numerosity in participant 1’s NTO map elicited by the large range. Recording points are binned based on preferred numerosity. Points represent the mean tuning width within each bin, error bars represent the standard errors of the mean over all the data points within each bin. Solid line is the linear fit to the bins, weighted by the inverse of the standard error of each bin. **b** Linear fits of tuning width against preferred numerosity of all the numerosity maps averaged across participants (solid coloured lines) and across maps (solid black line). In both (**a**) and (**b**): dashed lines represent 95% confidence intervals determined by bootstrapping fits to the binned points (*n* = 1000). The statistical significance of the slopes was determined with permutation analysis (*n* = 10,000), indicating the probability of observed tuning width change by chance.

To illustrate the change of tuning width with preferred numerosity, we plotted tuning width against preferred numerosity estimated by viewing the large numerosity range (Fig. 4). Population tuning widths increase with preferred numerosities systematically across all numerosity maps of all the participants (Supplementary Fig. 4), in line with the observation at the small numerosity range^27^. The cross validated datasets show similar changes of tuning width increase with preferred numerosity (Supplementary Fig. 5d, e).

## Discussion

We found a network of neural populations tuned to small and large numerosities organized as topographic maps in the same cortical regions. These neural populations exhibit stable numerosity selectivity regardless of presented numerosity range. When the participants were viewing the large range, i.e., 1–64, we found populations with larger numerosity preferences (above 7) located at the end of the maps (near higher preferences within the small range). These numerosity maps exhibit features akin to maps for primary sensory organs (retinotopic maps, tonotopic maps and somatotopic maps), such as a larger extent of cortical surface devoted to smaller numerosities, i.e., cortical magnification^34,35^. These results demonstrate a continuum of small and large numerosity preferences within the same numerosity map. We therefore propose a single neural mechanism for the ANS up to numerosities of 64. We suggest that small and large numerosities are encoded in the same neural tuning, nevertheless, small and large numerosities differ in their cortical representations. We speculate that the differences of the map properties, such as cortical magnification and tuning width, may underlie the different behavioural and perceptual qualities of small and large numerosities.

For the overlapping numerosities between the small and large ranges, i.e., 1–7, the numerosity maps were similar. When stimulating with larger numerosities (above 7), the numerosity maps extended in the direction of the higher preferences within the small range condition in a continuous fashion. This is akin to visual field maps, when stimulating with a greater eccentricity a larger proportion of the map is revealed^30^. Likewise, a wider numerosity range reveals a larger proportion of the numerosity map.

We propose that there are two main theories to explain the results. On one hand, we speculate that the numerosity tuning remains stable but that the stimulus range influences the numerosity responses. A single recording site (1.75 × 1.75 × 1.75 mm^3^) will have about 250,000 neurons^36^. In line with this notion, the tuning width of the total population within a single recording site is quite large: neural populations tuned to 2 have a tuning width of about 10 (see for example in Fig. 1g, h). Therefore, we assume that at a single neuron level, different preferred tunings are present in the same fMRI recording site, i.e., the population consists of neurons with different preferred numerosities. In other words, the heterogeneity of the neural population alters the overall numerosity preference depending on the presented range. More specifically, the overall numerosity preference of a recording site is an average of the preferred numerosities of the neural populations within this recording site. For example, at the same recording site, the averaged population tuning would be higher for the large numerosity range because the neurons sensitive to larger numerosities in the recording site will contribute more to the population responses when the larger numerosities are presented, and less when smaller numerosities are presented. We found only a slight increase of preferred numerosity at the same recording site (i.e., the slope is slightly above the unity line) when stimulated with the large range, even in the lower portion of the range (i.e., 1–7). However, the overall deviation is small (around 3.59%). This suggests that the majority of neurons within a recording site tend to have similar preferred numerosities. Furthermore, neural tuning estimated from the large range predicts a large signal variation of the responses derived from the small range, and vice versa (Fig. 2e). Therefore, we suggest that the numerosity preference of single neurons is likely stable, but the heterogeneity of the neural population may give rise to different preferred numerosity estimations when the stimulus changes.

On the other hand, another possible explanation is that the tuning of neural population depends on the presented stimuli and the numerosity maps are dynamic remapping of the tuning properties. Previous studies have demonstrated that numerosity is susceptible to adaptation akin to primary sensory perceptions^2,21,37^. Recently, Tsouli et al.^38^ found that numerosity adaptation altered the preferred numerosity within the numerosity map, resulting a predominantly attractive biases towards the numerosity of the adaptor. Moreover, the adaptation effect increases as the numerical distance between the unadapted preferred numerosity and the adaptor increases. Let us assume that the neural population at a recording site responded selectively to the numerosity 4. When stimulated repeatedly and sequentially with larger numerosities (e.g., 8–64), the preferred numerosity of the neural population could shift to a higher number, due to the attractive bias of adaptation towards the larger numerosities. Thus, the numerosity maps would show some systematic changes in numerosity preference depending on the numerosity range, i.e., dynamic remapping of the neural population tuning properties. As we note in the “Methods”, our stimulus sequence presented the numerosities changed systematically in both ascending and descending directions and the small and large ranges were interleaved during scanning. By doing so, we aim to balance opposing effects of preceding lower and higher numerosities and habituation effects of the small or large range. Furthermore, as Supplementary Fig. 1 shows, stimulating with only large numerosities (>7) resulted in poor estimates of the maps and only elicited responses at the maps consisting of neural populations tuned to larger numerosities. This suggests that the neural population tuning is less likely to change dynamically to follow the presented stimulus. Thus, though we cannot exclude context-depending remapping, we are not convinced of this theory given the possibility of range-dependent differences in the contributions of different parts of a heterogeneous neural population (the first theory). Therefore, we favour the interpretation that under our stimulus design the numerosity tuning remains predominantly stable.

In line with our findings, the stability of numerosity selectivity is also evident at a temporal scale. At a single neuron level, neurophysiological recordings on non-human primates demonstrated that numerosity-selective neurons maintain reliable tunings after the numerosity stimulations disappear^20,39^. Similarly, stable numerosity selectivity is also found in corvid birds when retaining information of numerosity in working memory and the neuronal activity during the delay period could predict behavioural performance^40^. These findings suggest that tuned responses of numerosity-selective neurons are stable across time, at least they hold information of the pre-presented numerosity in working memory. This enables a reliable neural system to maintain information temporally to deal with the task demand. Together with our findings, we suggest that numerosity tunings are stable, providing a reliable neural system for numerosity perception at the cortical representation and temporal processing scales.

The largest preferred numerosities detected in the numerosity maps were smaller than the largest presented numerosity (i.e., 64), and these neural populations are found located at the end of the map. In addition, stimulating with only larger numerosities (i.e., above 7) does not reveal the complete maps, or a clear topographic progression, but mainly produces responses at the sites where the maps have neural populations tuned to large numerosities (Supplementary Fig. 1c). There were few responses to larger numerosities beyond 12. This could be interpreted as evidence that the cortical encoding is different for larger numerosities than smaller ones. However, fewer responses to large numerosities does not necessarily mean there are no neurons responding to these large numerosities. Evidence from single neuron recordings demonstrate neurons selective for large numerosities^24^. In our study, neurons with tuning to very large numerosities may be hidden in the overall neural populational response dominated by neurons tuned to smaller numerosities. Therefore, we suggest that small and large numerosities are represented similarly in terms of their neural tunings.

Furthermore, based on our observations, less cortical area is devoted to representing larger numerosities. We assume that the largest numerosity we can measure is constrained by the surface area of the numerosity map. For example, the largest preferred numerosity of a given recording site (voxel) is derived by averaging the preferred numerosities of the neural populations within this site. In such a way, the representative preferred numerosity of a given recording site will always be smaller after averaging values from the subpopulations. This could also explain why the size of numerosity map correlates with the potential largest preferred numerosity within the map (Fig. 3d). If the map is small in size (fewer voxels), we cannot resolve individual populations preferring larger numerosities as they are mixed with those preferring smaller numerosities at the same recording sites. If the maps are larger in size however, we could distinguish the neural populations tuned to larger numerosities and those tuned to smaller numerosities in separated voxels.

Furthermore, we propose that the cortical magnification explains why stimulating with only larger numerosities (i.e., above 7) does not reveal the complete maps or topographic progression. We speculate that the cortical magnification factor, i.e., fewer cortical surface area is devoted to larger numerosities, accounts for the fact that fewer representations for larger numerosities (e.g., 16–64) were detected. In visual cortex, there is a smaller fraction of cortical surface for representing larger eccentricities^30,41^, likewise, there are evidences point at a similar decline in surface area for representing larger numerosities^27,28^. Thus, it seems likely that the detection of the largest numerosity was also constrained by the cortical magnification effect of the numerosity map representation. In support of this assumption, Cheyette et al.^42^ suggested that the limited amount of information processing capability of the underlying neural circuits leads to the inaccurate perception of large numerosity, while a single system represents small and large numerosity.

The continuum of cortical representation of small and large numerosities argues for a single numerosity neural representation mechanism, in line with the single enumeration system of the ANS. However, numerosity estimation is fast and accurate for the subitizing range, where some studies report a clear violation of Weber’s law^15,43^. Enumeration suddenly becomes slow and error-prone beyond this range, showing an increase in reaction time and a decrease in precision^44,45^. Therefore, this dissociation is held to reflect two separate systems in enumerations at different set sizes^13^. However, reported differences in the dependence for small versus large numbers do not necessary imply the existence of two separate systems. Because for small numerosities the imprecision of the numerosity representation remains below one item while for larger numerosities to achieve the same discrimination precision more numerical distance is required, which results in more overlap and a ratio-dependent effect^4,46^.

Although we suggest that a common neural mechanism underlies numerosity representation across a wide range, it may nevertheless have distinct perceptual and behavioural consequences between the subitizing and estimation ranges. The fast and accurate perception on small numerosities is because more cortical area of the numerosity maps are devoted to smaller numerosities^27,28^. This is consistent with the observation in macaque prefrontal cortex that single neurons with smaller numerosity preferences occurred more frequent, with a progressive decrease in frequency towards higher numerosity preferences^24^. This cortical representation of small and large numerosities resembles the logarithmic coding of numerosity^21,39,47^. Neurophysiological studies in macaque and corvids show logarithmically numerosity encoding in single neurons^32,40^. Logarithmic coding allows a wide range of numerosities to be encoded, thus increasing the scope of neural representation and perception of numerosity^48^. The cortical magnification of numerosity maps provides the neural circuits for such a logarithmic coding space. Perception on large numerosities gets inaccurate and takes more time as the tuning width increases with the preferred numerosity. Thus, we speculate that the properties of numerosity representation, such as cortical magnification and tuning width, give rise to distinct perceptual performance on small and large numerosities.

Despite much evidence for a number sense in humans, there have been arguments about whether numerosity is sensed directly or derived indirectly from other non-numerical information in the stimulus, such as dot size and density^49,50^. One reason why the argument is particularly compelling is that numerosity is intrinsically correlated with many other physical features. For example, we have shown a correlation between the neural tuning of object size and numerosity, with largely overlapping topographic maps. However, object size and numerosity tuning result from distinct mechanisms, indicated by their distinct tuning properties and map organizations^51^. Previous studies from other labs have demonstrated separate mechanisms for perception of numerosity and density^14,52^ that a regime of texture mechanism represents densely packed items that cannot be individuated as separate items. Note that in previous studies^27,28^, we used various stimulus conditions, such as constant area, constant dot size, constant circumference, high density and various shapes. In these studies, we consistently found topographic numerosity maps in all the stimulus configurations, which suggests that the topographic maps depend on numerosity rather than other stimulus information. We have also demonstrated that responses in these maps cannot be explained by neural tuning for these non-numerical features^53^. In the current study, we used a stimulus configurations total surface area held constant across numerosity, ensuring equal luminance in all the numerosity displays. The stimuli were presented in a larger central visual field of 4° than the original setting of 1.5°, as this configuration allows larger numerosity stimuli to have enough space to individuate each item. But in this stimulus configuration density increases with numerosity and total item perimeter decreases, for example. We believe the response we observe reflect numerosity because this has been conclusively demonstrated in the same maps in our previous studies^27,28,51^, although it was not possible to design the experiment to conclusively demonstrate this in the current data with the large numerosity range.

Based on these results, we suggest that differences in neural properties within the same topographic map underlie the different cognitive behaviours of numerosity perception. This is commonly seen in visual field maps with perceptual differences between central (foveal) and peripheral vision. Visual field maps show changes in cortical magnification and receptive field size with eccentricity. Specifically, more of the cortical area is devoted to central vision with smaller receptive fields. Such differences in cortical magnification and receptive field size may reflect different perceptual processing requirements^30^. Therefore, like visual cortex, we suggest that, not only are topographic maps a core principle of brain organization, but the differential features of cognitive topographic maps underlie differences in cognition.

## Methods

### Participants

We present data from eight participants (one female, age range 25–45 years). All the participants had normal or correct-to-normal visual acuity. All were well educated, with good mathematical abilities. Written informed consent was obtained before every MRI session. All experimental procedures were approved by the ethics committee of University Medical Centre Utrecht.

### Stimuli and experiment design

**Fig. 5:**
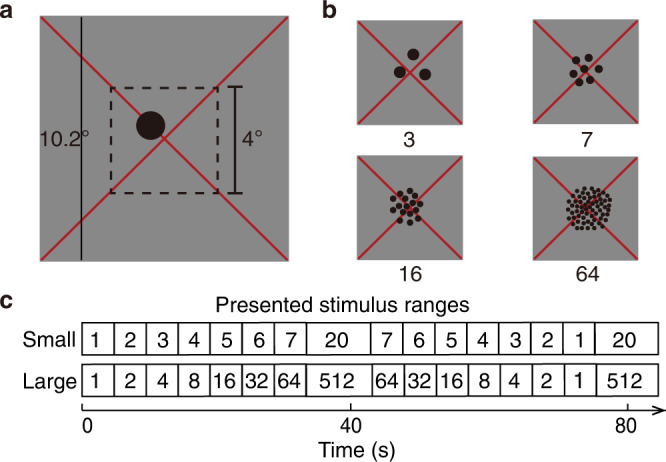
Illustration of stimuli and experimental design. **a** A full example stimulus as seen by the participants in the scanner. The dot pattern covered the central 4° (visual angle) diameter within an 10.2° diameter mean-luminance (grey) field. A large, thin, red fixation cross passes diagonally through the centre of the display, and through the centre of the dot pattern. Participants fixated at the intersection of the cross. **b** Example numerosity stimuli, where the total surface area of the dot pattern is constant across numerosities. **c** The sequence of the numerosity stimuli presented to the participants at the small and large ranges, respectively.

Visual stimuli were presented on a 69.84 × 39.29 cm LCD screen (Cambridge Research Systems) behind the MRI bore. Participant was required to lie still and view the display through a mirror attached to the head coil. The total distance from the attached mirror to the display screen was 220 cm. The display resolution was 1920 × 1080 pixels. Visual stimuli were generated in Matlab using PsychToolbox^54,55^. A large diagonal cross composed of thin red lines was displayed consistently across the entire screen, which allows accurate fixation. Participants were asked to fixate the intersection of the cross. Stimuli consisted of a group of dots with a constant total surface area presented in the central 4° (diameter) of the visual field. Dots were randomly positioned at each presentation so that each dot fell entirely within this area, to distribute contrast energy equally across the stimulus area for all numerosities (Fig. 5). Each numerosity presentation that contained the same number of dots was placed in a new, random position, so no specific visual position was associated with any numerosity. To prevent perceptual grouping, individual items were distributed roughly homogeneously across the stimulus area. All of the numerosity presentations were displayed as black or white dots on a grey background. Dot patterns were presented briefly (300 ms) to ensure participants did not have time to count. A new random pattern was presented every 650 ms, with 350 ms presentation of a uniform grey background between dot pattern presentations. This was repeated six times, over 3900 ms, corresponding to two fMRI volume acquisitions (TRs), before the numerosity changed. On 10% of numerosity presentations, the dots were shown in white instead of black. Participants were instructed to press a button when white dots were shown to ensure they were paying attention to the stimulus during the fMRI acquisition and responded to 90–100% of white dot presentations within each functional run. No numerosity judgements were required. Main stimuli in the small numerosity range consisted of 1 to 7 dots, with 20 dots as the baseline, while large numerosities consisted of 1 to 64 dots and a baseline of 512 dots. To test neural populations responses to larger numerosities, a third numerosity range consisted of only large numerosities from 8 to 64 dots and a baseline line of 512 dots was introduced, namely, the large-control range (Supplementary Fig. 1a). The main numerosity stimuli were first presented in ascending order, followed by a longer period (15.6 seconds) where presented with the baseline stimuli (20 or 512 dots in the small or large range respectively), then followed by the main numerosities in descending order, followed by another identical baseline period. This sequence was repeated four times (4 cycles) for each functional run. The long baseline period had a similar function to the blank periods used in visual field mapping stimuli in population receptive field experiments^33^. During this period, little neural response was expected from numerosity-selective neurons preferring the main numerosities of interest, as such a relatively large numerosity should be well outside of the numerosity range that elicits strong responses. This long period also allows hemodynamic responses to return to baseline between blocks of changing numerosity.

### MRI acquisition and preprocessing

Anatomical MRI data were acquired from a Philips 7 T scanner (Philips Medical Systems, Best, NL). MP2RAGE T1 anatomical MRI data were acquired at the spatial resolution of 0.64 × 0.64 × 0.64 mm^3^ (resampled to 1 × 1 × 1 mm^3^ for the follow-up processing), repetition time (TR) was 6.2 ms, echo time (TE) was 3 ms, and flip angle was 5/7 degrees. Functional T2*-weighted multi-band (factor=2) 2D echo planar images (EPI) were acquired on a Philips 7 T scanner using a 32 channel head coil (Philips Nova Medical) at a resolution of 1.75 × 1.75 × 1.75 mm^3^, with a full-brain-coverage field of view (FOV = 106 × 112 × 236) covering 64 slices. TR was 1950 ms, TE was 25 ms, and flip angle was 70 degrees. Functional runs were each 182 time frames (354.9 seconds) in duration, of which the first six time frames (11.7 s) were discarded to ensure the signal was at a steady state. Within each session eight functional runs were acquired with the small and large numerosity ranges interleaved to avoid adaptation. Each participant was scanned for two sessions on separate days. In addition, we collected eight functional runs on seven of our participants with the large-control range.

T1 anatomical scans were automatically segmented using CBS tools (www.nitrc.org) and then manually edited to minimize segmentation errors using ITK-SNAP^56^ (www.itksnap.org). This provides a highly accurate description of the cortical surface, an anatomical segmentation space used for analysis of cortical organization. The cortical surface was reconstructed at the grey-white matter border and rendered as a smoothed 3D surface. Head movement and motion artefacts between and within functional scans were measured and corrected for in AFNI^57^. Motion-corrected functional data were then averaged and the resulting mean image was co-registered to the segmented anatomy. Individual functional images were then co-registered to the same anatomical space using the same transformation.

### fMRI data analysis

Functional data analysis was performed in mrVista, which is freely available at (https://github.com/vistalab/vistasoft). First, data from separate sessions was imported into the same anatomical space for each participant. Functional runs (*n* = 8) collected for the same condition (small or large range) were averaged to produce a dataset with strong signal strength. Second, the averaged functional dataset was collapsed onto the nearest point on the cortical surface across depth to further increase on signal strength, which generated a (folded) 2D grey matter surface. Then we performed the canonical numerosity modelling developed to estimate the tuning properties of numerosity-selective neural populations^27,33^. Briefly, a one-dimensional neural model defined as a Gaussian function in logarithmic space was adopted. The Gaussian function characterized by a set of parameters: preferred numerosity (mean) and tuning width (standard deviation). The model predicts neural responses by taking the presented numerosity at each time point and evaluating the Gaussian function’s amplitude at this numerosity. Then convolving these predicted neural response time course with a hemodynamic response function (HRF) to generate predicted fMRI time courses. The predicted fMRI time course with the minimum sum of squared errors (R^2^) residuals to the recorded signal was chosen, and the Gaussian function parameters that generated this prediction were used to summarize the recording site’s response. The goodness of model fit (R^2^, i.e. variance explained) was thresholded at 30% to select recording sites with clear numerosity selective responses: recording sites with lower variance explained were excluded from further analysis. The modelling procedure allows preferred numerosity estimates outside the range of the presented stimuli, ensuring estimates within the stimulus range are not just the best of a limited set. We excluded from analysis any recording sites where the preferred numerosity was outside the presented range accordingly. Finally, the preferred numerosity data was projected onto the smoothed cortical surface.

### Definition of region of interest

We defined regions of interest (ROI) where the numerosity-selective neural populations are organized topographically similar to previously reported numerosity maps^28^. In total, six ROIs were drawn for the right hemisphere in the temporo-occipital cortex, parieto-occipital cortex, parietal cortex, and superior frontal cortex, corresponding to six numerosity maps: NTO, NPO, NPC1, NPC2, NPC3, NF. In each ROI, we defined map borders on the lowest and highest preferred numerosities (white lines) and the map edges around the local increase in model goodness of fit (black lines) (Fig. 1a, b, Supplementary Fig. 1b).

### Correlation analysis between numerosity preferences

Pearson correlation analysis was performed between numerosity preference estimated from the small and large ranges. This included the recording sites that had variance explained above 30% in both conditions. Taking into account the functional resolution of the recording sites, the total number of data points (n) used to calculate correlation’s probability was reduced by the factor by which functional voxels were up-sampled onto the 2D cortical surface.

To quantify the similarity between the numerosity preferences estimated from the two ranges, we calculated the percentage deviation. We calculated the difference of the slopes between the linear fit line of the numerosity preference correlation and the unity line (*y* = *x*). The percentage deviation of the unity line was set to 0, indicating that the estimates of small and large numerosity preference are equal. The largest possible deviation is indicated by the best fit function of *y* = 10.5*x*−9.5, where the estimate of the largest numerosity at small range (i.e. 7) corresponds to the estimate of the largest numerosity at the large range (i.e., 64). The percentage deviation of this best possible fit was set to 1. Thus, for each map, the percentage deviation = (*p*-1)/9.5, where *p* is the slope of the best fit of the correlation. We performed a Wilcoxon signed rank test (two-tailed) to the percentage deviations of all maps in all participants. A two-way ANOVA was performed to test the statistical difference in the percentage deviations between maps and participants, followed by post hoc analysis with Bonferroni correction for multiple comparisons.

### Analysis of change of numerosity preferences along maps

For each ROI, we calculated the distance of each recording site to the nearest points on the borders of the map with the lowest and highest numerosity preferences. The ratio between the distances to each border was computed, which gives a normalized distance along the ROI in the primary direction of preferred numerosity change. Then we multiplied this normalized distance by the mean length of the ROI in this direction, which gives a measure of the distance along the ROI for each recording site.

We binned the data points within every 2 mm distance interval along each ROI. The mean and standard error of the preferred numerosity of the points within the bin was calculated. We fitted logarithmic functions to bootstrapped samples of the bin means. From these bootstrapped fits we took the median slope and intercept as the best fitting numerosity progression. We determined 95% confidence intervals by plotting all lines generated during bootstrapping iterations and finding the 2.5 and 97.5 percentiles values for these fits. The statistical significance of the slopes was determined with a permutation analysis, where the order of distance bins was randomized (10,000 times). The slopes were fitted at each permutation, and the probability of finding the observed slope by chance was calculated as the number of times where the slope in the randomized permutation was equal to or greater than the observed slope.

We normalized the cortical distance of each ROI to visualize the progression of numerosity preference in a similar way. We binned the recording sites within every 10% interval of the normalized cortical distance along each ROI. To visualize the location of neural populations selectively responding to larger numerosities (above 7), we sorted neural populations preferred large numerosities into three subranges (i.e., 7–16; 16–32; 32–64) at each bin. We calculated the proportion of these recording sites among all the selected recording sites in the same bin. The proportions of each subrange at each bin of all maps in all participants were averaged and stacked. Last, we extracted the largest preferred numerosity of each map estimated from the large range and calculated the correlation between these preferred numerosities and the cortical distance of the maps.

### Analysis of change of tuning width with numerosity preference

In each ROI, we binned data based on preferred numerosities at each range, with numerosity increments of 0.25 between bins. The mean and standard error of each bin were calculated. We fitted linear functions to bootstrapped samples of the bin means. We determined 95% confidence intervals by plotting all lines generated during bootstrapping iterations and finding the 2.5 and 97.5 percentiles values for these fits. Similar permutation analysis, as described above, was used to calculate the probability of finding the observed tuning width change by chance. Unstable fits are common seen in some ROIs where there are little information in the data set to distinguish tuning widths.

### Cross validation analysis

We cross validated the results by splitting the data into two halves for each condition, based on odd versus even runs, resulting in four half cross validation datasets (i.e., small-odd, small-even, large-odd and large-even). Two types of cross validations were done: within-condition and cross-condition. We selected the recording points from each cross validation datasets based on the criterion that the preferred tuning from 1 to 7, which present at both the small and the large ranges.

For the within-condition validation, we extracted the model prediction of the selected voxels from one dataset (e.g., large-odd) and fitted that to the other dataset (e.g., large-even) of the same condition and vice versa, namely the “small → small” and “large → large” validations. This resulted in two iterations of each condition and we calculated the cross-validated variance explained (cvR^2^) of each iteration. For the cross-condition validation, we extracted the model prediction from one cross validation dataset (e.g., small-odd) and fitted that to the two datasets of a different condition (e.g., large-odd & large-even), namely the “small → large” and “large → small” validations. This resulted in eight iterations of cross validation by taking the model prediction from each dataset in turn. We then calculated the averaged within-condition and cross-condition cvR^2^ across all iterations and across maps and participants. A within-subject repeated measures two-way ANOVA analysis was performed using JASP to compare within- and cross-condition validations (Fig. 2e)^58^.

To validate the results of the large range data, we selected the voxels with the criteria that the preferred tuning fell at the large range and with the cvR^2^ larger than 30%. We replicated the main analyses using the cross validation datasets (see Supplementary Fig. 5).

### Reporting summary

Further information on research design is available in the Nature Research Reporting Summary linked to this article.

## Supplementary information

Supplementary Information

Reporting Summary

## Data Availability

The data sets generated during the current study are available from the corresponding author upon reasonable request. Source data of presented figures are provided with this paper. Source data are provided with this paper.

## Source data

Source Data

## Code availability

The code that supports the findings of this study is available from the corresponding author upon reasonable request.
