# Supplementary material for: Topographic numerosity maps cover subitizing and estimation ranges
Source: Nat Commun. 2021 Jun 7;12:3374. doi: 10.1038/s41467-021-23785-7 (PMC8184945; doi:10.1038/s41467-021-23785-7)
Supplement: Supplementary file 1 — Supplementary Information [file 41467_2021_23785_MOESM1_ESM.pdf]

Topographic numerosity maps cover subitizing and estimation ranges

Supplementary Information

Yuxuan Cai[1,2*], Shir Hofstetter[1], Jelle van Dijk[1], Wietske Zuiderbaan[1], Wietske van der Zwaag[1], Ben M. Harvey[3], Serge O. Dumoulin[1,2,3*]

1. Spinoza Centre for Neuroimaging, Amsterdam, Netherlands

2. Experimental and Applied Psychology, VU University Amsterdam, Amsterdam, Netherlands

3. Experimental Psychology, Helmholtz Institute, Utrecht University, Utrecht, Netherlands

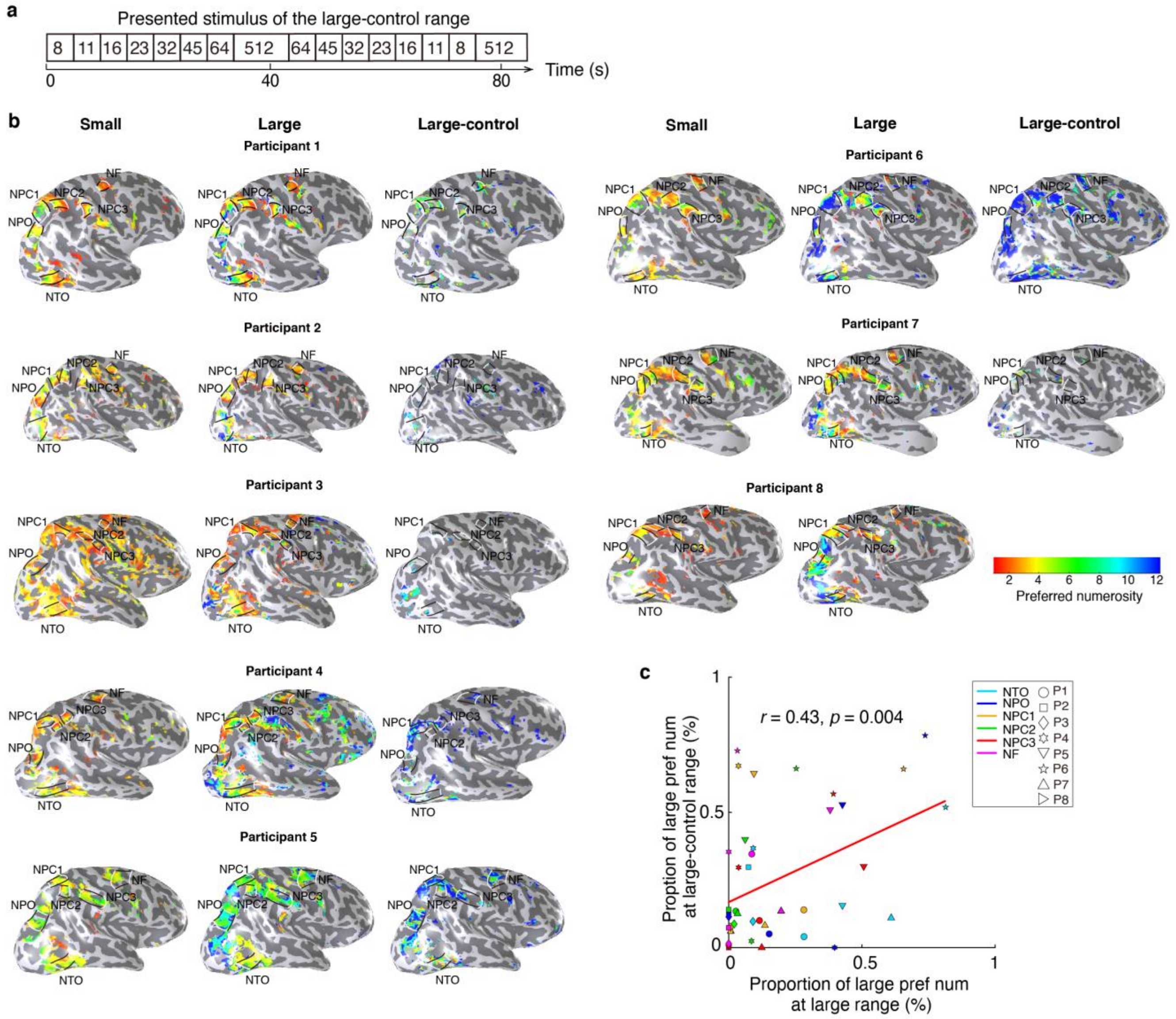

Supplementary Figure 1. Numerosity maps of all participants and relationship of large numerosity preferences at the large and large-control ranges. **a** Illustration of the large-control range. **b** Numerosity maps of the small, large and large-control ranges. Cortical surface rendering of the right hemisphere of all the participants shows a constant and similar network of numerosity maps at both the small and the large ranges. However, stimulating only with large numerosities (>7, panel **a**) reveals only part of the maps. Preferred numerosities are shown where the model explained over 30% of response variance within the recording site. Black lines outline individual numerosity maps. The boarders of the lowest to the highest preferred numerosity in each map are marked by white lines. **c** Numerosity maps with more neural populations tuned to large numerosities have more responses elicited by the large-control range. Given the cortical magnification, numerosity maps show few responses to large numerosities and thus, most of the maps show little responses to the large-control range. Source data are provided as a source data file.

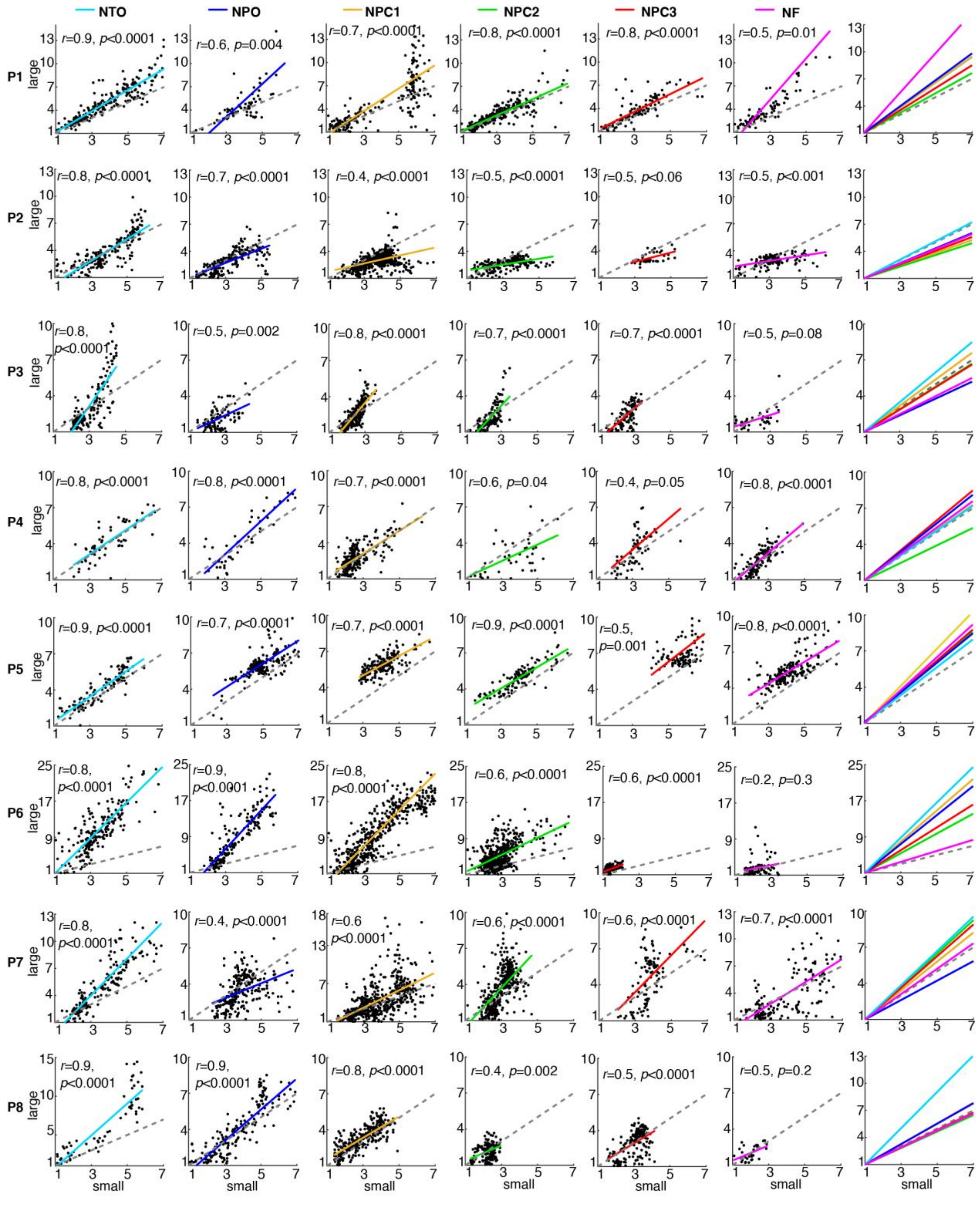

Supplementary Figure 2. Correlations between numerosity preferences estimated from small and large ranges indicate similar numerosity selectivity among individual maps and participants (P1–P8). Dots show the estimates from individual recording sites (variance explained > 30%), coloured lines indicate the best linear fits between the two estimates, the dashed line shows unity (i.e. identical estimates). Texts in legends indicate the Pearson correlation coefficients (*r*) and statistical significance (*p*).

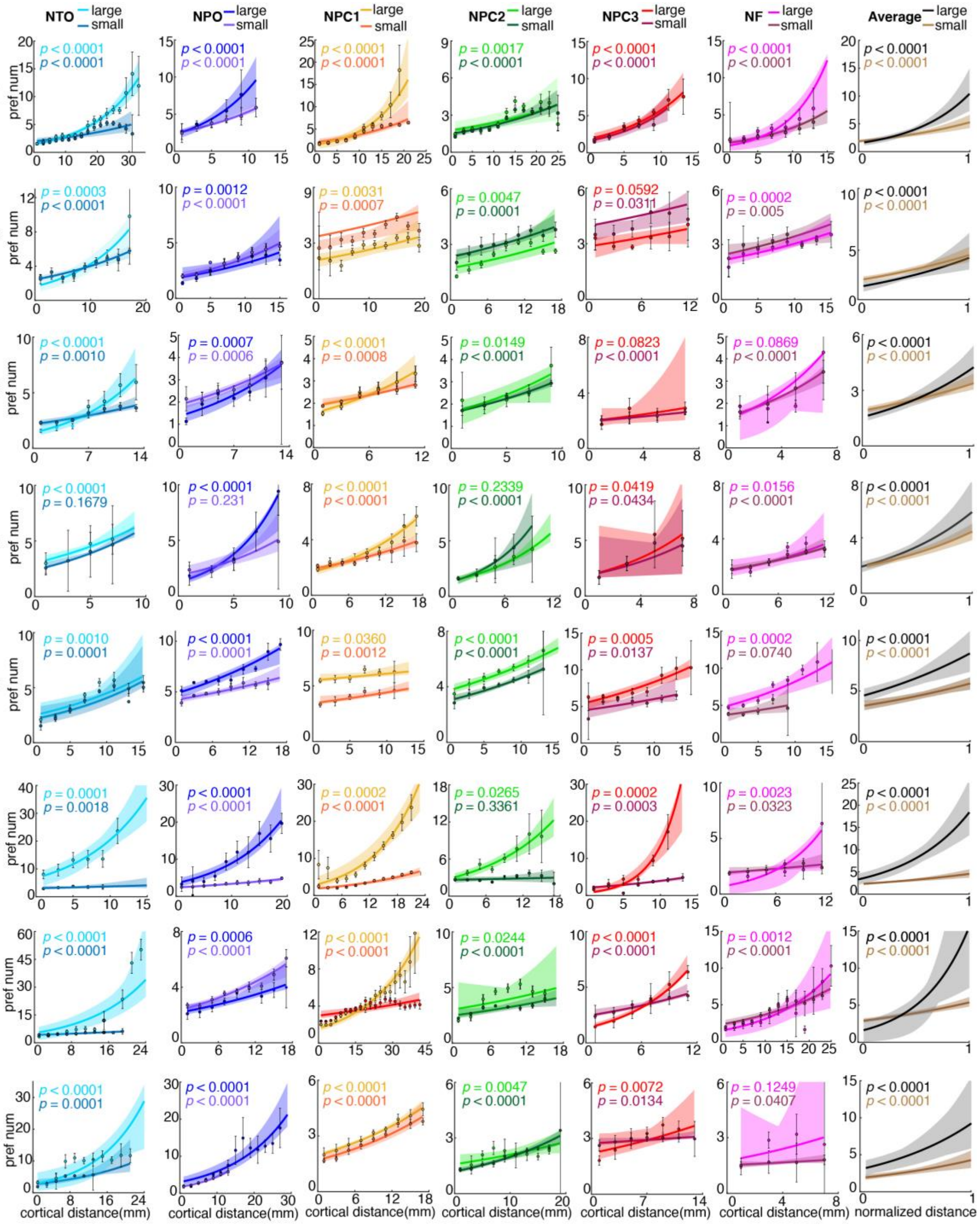

Supplementary Figure 3. Cortical progression of small and large preferred numerosities with the cortical distance in individual maps, and averaged across maps against the normalized cortical distance (right panel), in all the participants (P1–P8). Preferred numerosities increase systematically for both conditions. Points represent the mean preferred numerosity in each distance bin (every 2mm interval), with error bars showing the standard errors of the mean over all data points with each bin. Coloured lines show the best logarithmic fits. Shade area shows the 95% confidence interval determined by bootstrapping fits (n = 1,000) to the binned points and p-values indicate the probability of the observed change from permutation analysis (n = 10,000).

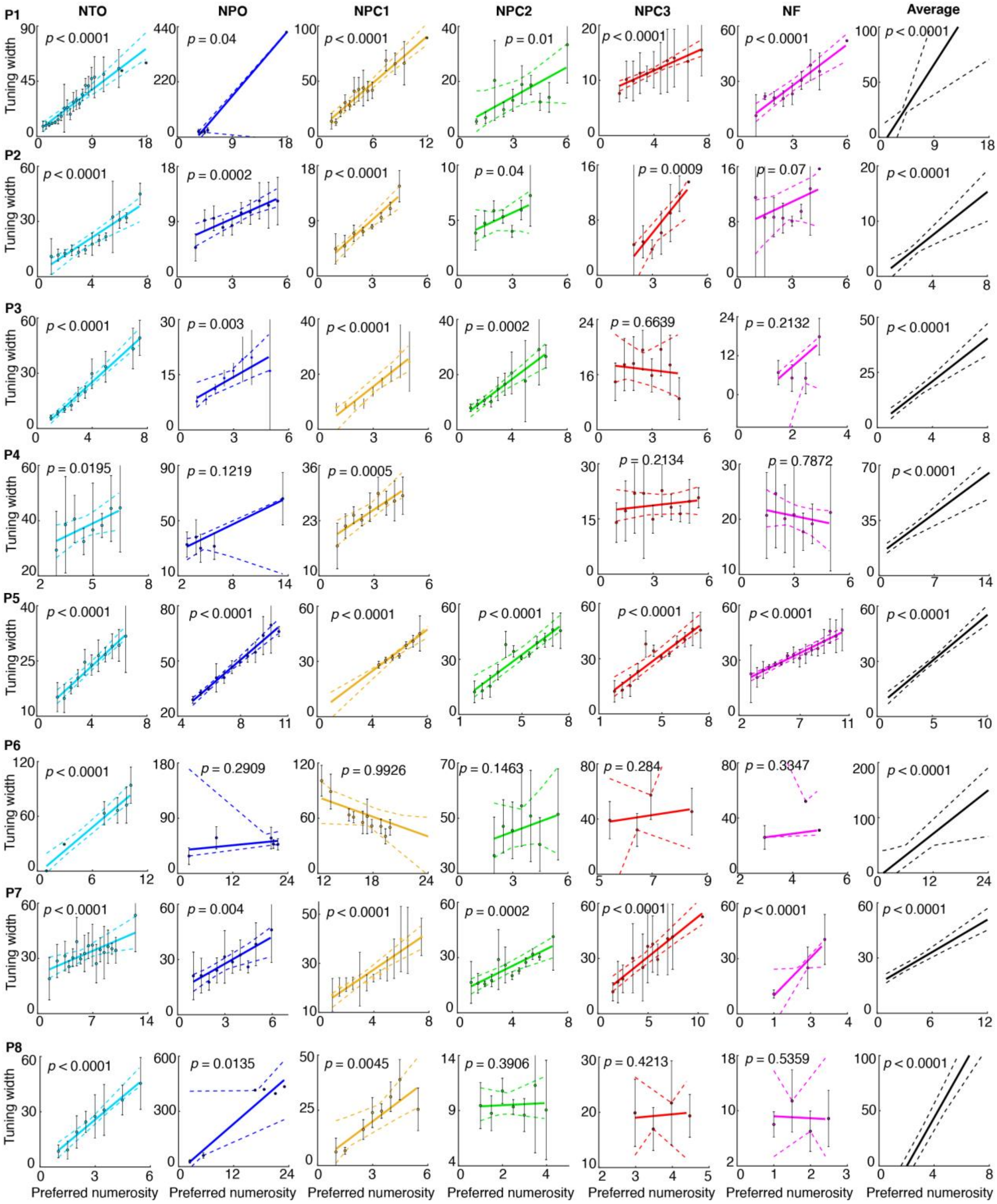

Supplementary Figure 4. Tuning width changes with preferred numerosity of all maps and all participants. Points represent the mean tuning width in each bin, error bars represent the standard error of the mean over all data points in each bin. Solid lines are the linear fit to the bins, weighted by the inverse of the standard error of each bin. Dashed lines represent 95% confidence intervals determined by bootstrapping fits to the binned points (n = 1,000). P-values give the probability of the observed change from permutation analysis (n = 10,000).

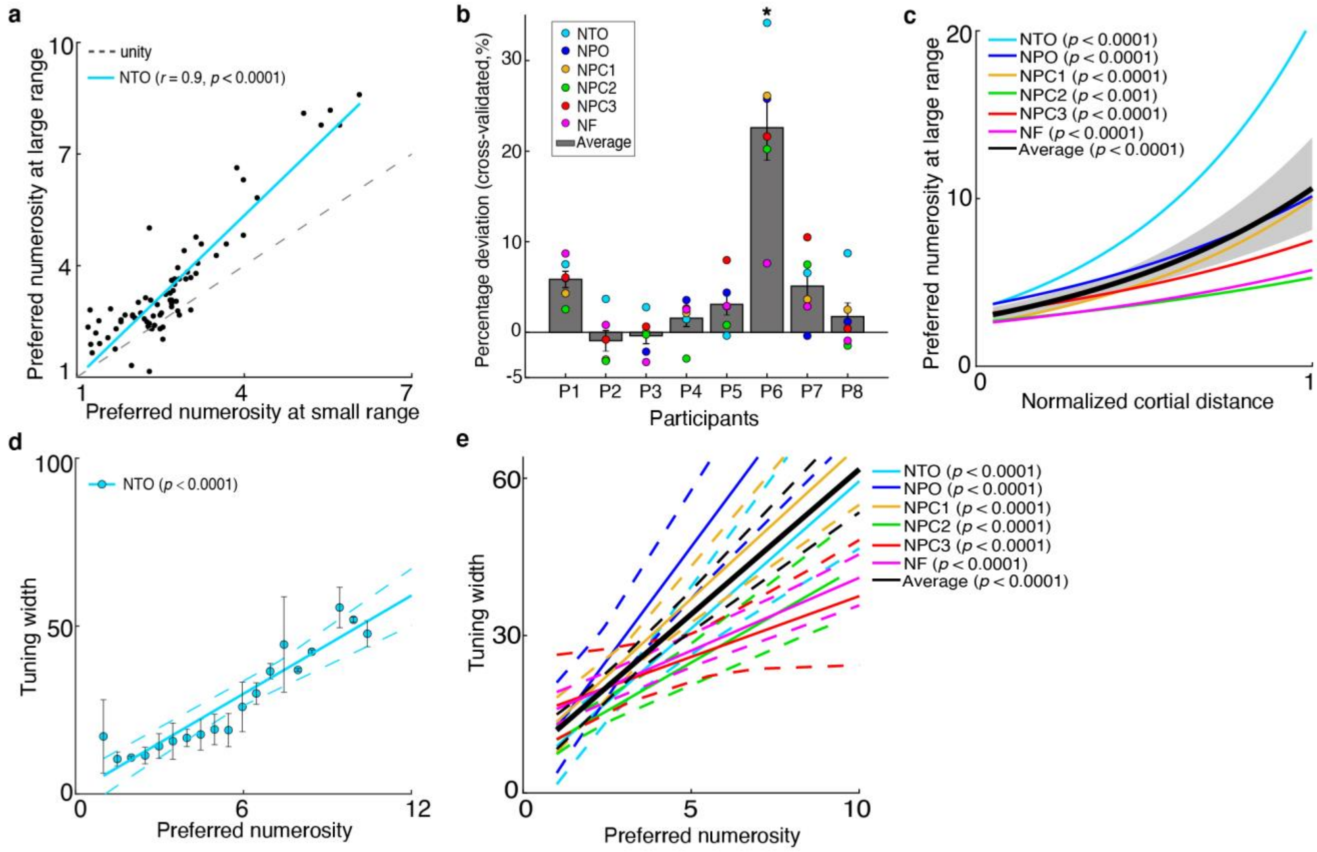

Supplementary Figure 5. The cross-validation datasets show near identical results. **a** Participant1's NTO numerosity preferences estimated from one pair of the cross-validation datasets (small-odd vs. large-odd) were strongly correlated (see legend for the Pearson correlation coefficient and statistical significance). Dots show the estimates from individual recording sites with a variance explained > 30% across all iterations (n = 8). Blue line shows the linear fit between the two estimates; dashed line shows unity (i.e. identical preferences). **b** Bars show the mean cross-validated percentage deviations for each participant; error bars show the standard errors of the mean over maps (n = 6). Only participant 6 has a significant higher percentage deviation from the unity line than other participants (two-way ANOVA analysis, followed by post hoc analysis, Bonferroni corrected for multiple comparison; $F_{(7,47)}$ = 23.1, * indicates $p = 2.3 \times 10^{-11}$). **c** Progression of numerosity preferences estimated from the split dataset of the large range as a function of normalized cortical distance in all numerosity maps, across all participants. The black line shows the best logarithmic fit of the bins from all the maps across normalized cortical distance. Shade area shows the 95% confidence interval determined by bootstrapping fits (n = 1,000) to the binned points and p-values indicate the probability of the observed change from permutation analysis (n = 10,000). **d** Tuning width increases with preferred numerosity in participant 1's NTO map averaged across the two split datasets of the large range. Recording points were divided into bins based on preferred numerosity. Points represent the average of the mean tuning width within each bin across the two split datasets, error bars represent the standard errors of the mean over the data points in each bin across the two split datasets. Solid line is the best linear fit, weighted by the inverse of the standard deviation of each bin. **e** Linear fits of tuning width against preferred numerosity of all the numerosity maps averaged across the two splits

of the large range, across participants (coloured lines) and across maps (black line). In both panel **d** & **e**: dashed lines represent 95% confidence intervals of the fits (coloured lines) to the binned points determined by bootstrapping (n = 1,000). P-value give the probability of the observed change from permutation analysis (n = 10,000). Source data are provided as a source data file.
