# Supplementary material for: Topographic numerosity maps cover subitizing and estimation ranges
Source: Nat Commun. 2021 Jun 7;12:3374. doi: 10.1038/s41467-021-23785-7 (PMC8184945; doi:10.1038/s41467-021-23785-7)
Supplement: Supplementary file 2 — Reporting Summary [file 41467_2021_23785_MOESM2_ESM.pdf]

nature research

Corresponding author(s): Yuxuan Cai

Last updated by author(s): Apr 13, 2021

Reporting Summary

Nature Research wishes to improve the reproducibility of the work that we publish. This form provides structure for consistency and transparency in reporting. For further information on Nature Research policies, see our Editorial Policies and the Editorial Policy Checklist.

Statistics

For all statistical analyses, confirm that the following items are present in the figure legend, table legend, main text, or Methods section.

n/a	Confirmed	
☐	☒	The exact sample size (*n*) for each experimental group/condition, given as a discrete number and unit of measurement
☐	☒	A statement on whether measurements were taken from distinct samples or whether the same sample was measured repeatedly
☐	☒	The statistical test(s) used AND whether they are one- or two-sided<br>*Only common tests should be described solely by name; describe more complex techniques in the Methods section.*
☐	☒	A description of all covariates tested
☐	☒	A description of any assumptions or corrections, such as tests of normality and adjustment for multiple comparisons
☐	☒	A full description of the statistical parameters including central tendency (e.g. means) or other basic estimates (e.g. regression coefficient) AND variation (e.g. standard deviation) or associated estimates of uncertainty (e.g. confidence intervals)
☐	☒	For null hypothesis testing, the test statistic (e.g. F, t, r) with confidence intervals, effect sizes, degrees of freedom and P value noted<br>*Give P values as exact values whenever suitable.*
☒	☐	For Bayesian analysis, information on the choice of priors and Markov chain Monte Carlo settings
☒	☐	For hierarchical and complex designs, identification of the appropriate level for tests and full reporting of outcomes
☐	☒	Estimates of effect sizes (e.g. Cohen's *d*, Pearson's *r*), indicating how they were calculated

Our web collection on statistics for biologists contains articles on many of the points above.

Software and code

Policy information about availability of computer code

Data collection	Philips Achieva 7.0 Tesla running on R5.1.7; PsychToolbox-3;
Data analysis	Matlab version 2018b (The Mathworks), AFNI (version 17.0.13), CBS tools (v7.4.0), ITK-SNAP (version 1.6.0.1), mrVista (version 2.1, https://github.com/vistalab/vistasoft); JASP(version 012.2.0)

For manuscripts utilizing custom algorithms or software that are central to the research but not yet described in published literature, software must be made available to editors and reviewers. We strongly encourage code deposition in a community repository (e.g. GitHub). See the Nature Research guidelines for submitting code & software for further information.

Data

Policy information about availability of data

All manuscripts must include a data availability statement. This statement should provide the following information, where applicable:

- Accession codes, unique identifiers, or web links for publicly available datasets
- A list of figures that have associated raw data
- A description of any restrictions on data availability

All data presented in this paper are stored in institute computers and are available upon reasonable request. Source data of presented figures are provided with this paper.

Field-specific reporting

Please select the one below that is the best fit for your research. If you are not sure, read the appropriate sections before making your selection.

☒ Life sciences ☐ Behavioural & social sciences ☐ Ecological, evolutionary & environmental sciences

For a reference copy of the document with all sections, see nature.com/documents/nr-reporting-summary-flat.pdf

Life sciences study design

All studies must disclose on these points even when the disclosure is negative.

Sample size	Eight healthy individuals participated in this study. Each participant was scanned for two sessions of the small and large range conditions. Seven out of these eight participants were scanned for a third session of the large-control condition. Thus, eight 7T fMRI runs were collected for each condition of each participant.<br>Sample size was chosen based previous papers (Harvey et al., Science 2013; PNAS 2015; Nature Human Behavior 2017). Our analyses aim to show clear results at the individual participant level. Consistent results in terms of the similar cortical locations of the numerosity maps were found across participants, indicating that the results can be generalized.
Data exclusions	After the fit of the numerosity model we excluded the data points that having the variance explained lower than 30% and those with the preferred numerosities outside the presented range at each condition, respectively. Though not pre-established, these criteria of data exclusion were in line with previous papers (Harvey et al., Science 2013; PNAS 2015; Nature Human Behavior 2017).
Replication	The results of one of the conditions is a replication of previous findings (Harvey et al., Science, 2013). We replicated the results across individual participants. We performed cross validation analyses by splitting the data into two haves. The results of cross validation data were near identical to the main results.
Randomization	The order of the conditions was randomized among the participants and scanning sessions.
Blinding	The investigators were not blind to group allocation. However, the identity of the participants does not have any effect on data collection. The investigators were blind to the participants identity during data analyses.

Reporting for specific materials, systems and methods

We require information from authors about some types of materials, experimental systems and methods used in many studies. Here, indicate whether each material, system or method listed is relevant to your study. If you are not sure if a list item applies to your research, read the appropriate section before selecting a response.

Materials & experimental systems

n/a	Involved in the study	
☒	☐	Antibodies
☒	☐	Eukaryotic cell lines
☒	☐	Palaeontology and archaeology
☒	☐	Animals and other organisms
☐	☒	Human research participants
☒	☐	Clinical data
☒	☐	Dual use research of concern

Methods

n/a	Involved in the study	
☒	☐	ChIP-seq
☒	☐	Flow cytometry
☐	☒	MRI-based neuroimaging

Human research participants

Policy information about studies involving human research participants

Population characteristics	We presented data from eight participants. One female, age range 25 - 45.
Recruitment	All participants volunteered to take part in the experiments. The participants were recruited through the research facility. Results of the experiment are not expected to be biased based on sample selection or participants knowledge of the experimental aims or design.
Ethics oversight	All experiments were approved by the ethic committee at University Medical Centre Utrecht.

Note that full information on the approval of the study protocol must also be provided in the manuscript.

Magnetic resonance imaging

Experimental design

Design type	Task fMRI; population receptive field (pRF) design
Design specifications	We collected two sessions per participant in separate days. Each session had eight runs of the same condition. Condition order was randomized among participants. There were four cycles within each run. Main stimuli in the small numerosity range consisted of 1 to 7 dots, with 20 dots as the baseline, while large numerosities consisted of 1 to 64 dots and a baseline of 512 dots. The main numerosity stimuli were first presented in ascending order, followed by a longer period (15.6 seconds) where presented with the baseline 20 stimuli (20 or 512 dots in the small or large range respectively), then followed by the main numerosities in descending order, followed by another identical baseline period. To test neural populations responses to larger numerosities, a third numerosity range consisted of only large numerosities from 8 to 64 dots and a baseline line of 512 dots was introduced, namely, the large-control range.
Behavioral performance measures	In 10% of the numerosity presentations, the dots were shown in white instead of black. Participants were required to press a button when white dots were shown to ensure they were paying attention to the stimuli during fMRI acquisition. We recored the percentage of correct responses. No numerosity judgments were required.

Acquisition

Imaging type(s)	functional and structual MRI
Field strength	7 Tesla
Sequence & imaging parameters	MP2RAGE T1 anatomical MRI data were required at the spatial resolution of 0.64 x 0.64 x 0.64 mm3, TR = 6.2ms, TE = 3ms, FA = 5/7degree.<br>Functional T2*-weighted multi-band (factor =2) 2D GE EPI were required on a Philips 7T scanner using a 32 channel head coil at a resolution of 1.75 x 1.75 x 1.75 mm, with a FOV = 106 x 112 x 236 covering 64 slices. TR = 1950ms, TE=25ms, FA=70 degree.
Area of acquisition	Whole brain coverage scan

Diffusion MRI ☐ Used ☒ Not used

Preprocessing

Preprocessing software	AFNI (version 17.0.13) for motion correction; CBS tools (v7.4.0) & ITK-SNAP (version 1.6.0.1) for anatomical segmentation; mrVista (version 2.1, https://github.com/vistalab/vistasoft) for aligning and transforming functional data to the anatomy.
Normalization	No normalization.
Normalization template	Data were performed in the native space.
Noise and artifact removal	Data was motion-corrected. No spatial and temporal smoothing was applied.
Volume censoring	First six volumes of each functional run were discarded. No other volume censoring was applied.

Statistical modeling & inference

Model type and settings	Population receptive field model. The properties of numerosity-selective neural populations were modeled using a one-dimension logarithmic Gaussian function defined with two parameters, i.e., preferred numberosity (center position) and tuning width (full-width-half-maximum).
Effect(s) tested	We compared the model parameters using linear regression and ANOVA analyses.

Specify type of analysis: ☒ Whole brain ☐ ROI-based ☐ Both

Statistic type for inference (See Eklund et al. 2016)	Voxel-wise
Correction	The voxel-wise pRF model fits were not corrected for multiple comparisons, but boostrapping and permutation analyses were used for all the following statistics on the numerosity maps.

Models & analysis

n/a	Involved in the study
☒	☐ Functional and/or effective connectivity
☒	☐ Graph analysis
☐	☒ Multivariate modeling or predictive analysis

Multivariate modeling and predictive analysis	pRF modeling
